# Franking of Vaccine Virus

**Published:** 1860-06

**Authors:** 


*Franking of Vaccine Virus*—The London *Lancet* is endeavoring to induce the authorities in Great Britain to consider the propriety of permitting the free transmission of vaccine matter by post.

Although the cost of mailing is there, as here very slight, yet everything which facilitates the transmission of fresh vaccine matter throughout the country, is of importance.—*Reporter.*

